# Pesticide exposure and risk of aggressive prostate cancer among private pesticide applicators

**DOI:** 10.1186/s12940-020-00583-0

**Published:** 2020-03-05

**Authors:** Larissa A. Pardo, Laura E. Beane Freeman, Catherine C. Lerro, Gabriella Andreotti, Jonathan N. Hofmann, Christine G. Parks, Dale P. Sandler, Jay H. Lubin, Aaron Blair, Stella Koutros

**Affiliations:** 1 Occupational and Environmental Epidemiology Branch, Division of Cancer Epidemiology and Genetics, National Cancer Institute, 9609 Medical Center Drive, Rockville, MD 20850 USA; 2 National Institute of Environmental Health Sciences, 111 T.W. Alexander Drive, Research Triangle Park, NC 27709 USA; 3 Biostatistics Branch, Division of Cancer Epidemiology and Genetics, National Cancer Institute, 9609 Medical Center Drive, Rockville, MD 20850 USA; 4 Occupational and Environmental Epidemiology Branch, Division of Cancer Epidemiology and Genetics, National Cancer Institute, 9609 Medical Center Dr., Room #6E616, MSC 9771, Bethesda, MD 20892 USA

**Keywords:** Pesticide, Aggressive prostate cancer (PCa), Organodithioate insecticides, Pesticide applicators

## Abstract

**Background:**

Prostate cancer (PCa) is one of the most commonly diagnosed cancers among men in developed countries; however, little is known about modifiable risk factors. Some studies have implicated organochlorine and organophosphate insecticides as risk factors (particularly the organodithioate class) and risk of clinically significant PCa subtypes. However, few studies have evaluated other pesticides. We used data from the Agricultural Health Study, a large prospective cohort of pesticide applicators in North Carolina and Iowa, to extend our previous work and evaluate 39 additional pesticides and aggressive PCa.

**Methods:**

We used Cox proportional hazards models, with age as the time scale, to calculate hazard ratios (HRs) and 95% confidence intervals (CIs) for the association between ever use of individual pesticides and 883 cases of aggressive PCa (distant stage, poorly differentiated grade, Gleason score ≥ 7, or fatal prostate cancer) diagnosed between 1993 and 2015. All models adjusted for birth year, state, family history of PCa, race, and smoking status. We conducted exposure-response analyses for pesticides with reported lifetime years of use.

**Results:**

There was an increased aggressive PCa risk among ever users of the organodithioate insecticide dimethoate (*n* = 54 exposed cases, HR = 1.37, 95% CI = 1.04, 1.80) compared to never users. We observed an inverse association between aggressive PCa and the herbicide triclopyr (*n* = 35 exposed cases, HR = 0.68, 95% CI = 0.48, 0.95), with the strongest inverse association for those reporting durations of use above the median (≥ 4 years; *n* = 13 exposed cases, HR=0.44, 95% CI=0.26, 0.77).

**Conclusion:**

Few additional pesticides were associated with prostate cancer risk after evaluation of extended data from this large cohort of private pesticide applicators.

## Introduction

Prostate cancer (PCa) is common among men in developed countries, however, little is known about modifiable risk factors [1]. Investigating potential risk factors for prostate cancer is challenging because incidence rates are affected by PCa screening. Thus, to avoid potential detection bias, epidemiologic analyses often limit evaluations of prostate cancer to clinically relevant subtypes [2].

Previous epidemiologic studies have linked farming to an increased risk of prostate cancer [3–9]. Analyses of data from the Agricultural Health Study (AHS) revealed a significant excess of both PCa incidence [10] and mortality [11] among pesticide applicators compared to the general population. Exposure to specific individual organochlorine (OC) and organophosphate (OP) insecticides have been linked to prostate cancer in multiple studies [12–16]. Specifically, a previous evaluation in the AHS reported increased risks of aggressive PCa with exposure to aldrin (OC) as well as the organodithioate class of OP insecticides, including fonofos (OP), terbufos (OP), and malathion (OP) [12]. Other studies reported associations between increased risks of prostate cancer and chlordecone (OC) [15, 16] as well as serum metabolite concentrations of chlordane (OC) [13], hexachlorocyclohexanes (OC) [14], and DDT (OC) [14, 16].

We previously published analyses on exposure to 50 commonly reported pesticides used at and before study enrollment and risk of aggressive PCa in the AHS [12]. In the current paper, we use data from the AHS to evaluate possible associations between aggressive PCa and the use of 39 additional pesticides not previously considered by adding 13 years of follow-up time and 811 additional aggressive PCa cases.

## Methods

### Study population and case ascertainment

**Fig. 1:**
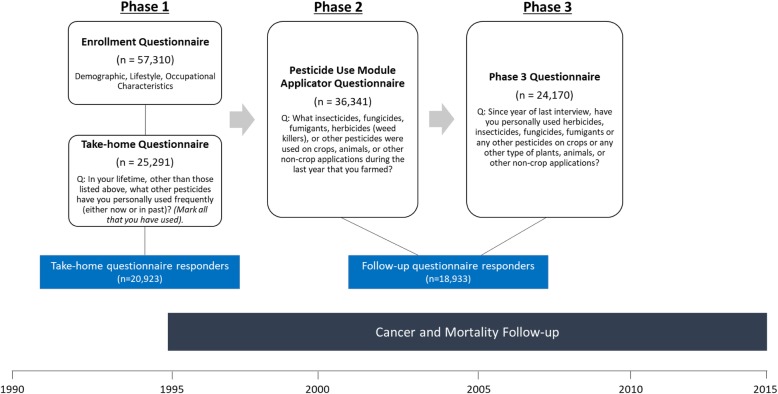
Timeline of study phases, questionnaires, and follow-up for the Agricultural Health Study (AHS) along with analytic populations for the current study

The AHS is an ongoing prospective cohort that includes 52,934 licensed private pesticide applicators in Iowa and North Carolina and 4916 licensed commercial applicators in Iowa. The cohort has been described in detail previously [17]. Briefly, the cohort is composed of individuals (82% of the target population enrolled) seeking licenses for pesticides which the U.S. Environmental Protection Agency (EPA) designated restricted use. In the AHS, pesticide exposure information has been collected in 3 phases of questionnaires--Phase 1 (1993–1997), Phase 2 (1999–2003), and Phase 3 (2005–2010),—with each phase including self-administered questionnaires or a computer assisted telephone interview (CATI) covering demographic, lifestyle, and occupational characteristics. In Phase 1, a total of 57,310 applicators completed the enrollment questionnaire and 25,291 returned the ‘Take-Home Applicator’ questionnaire. Of those initially enrolled, 36,341 applicators completed the Phase 2 ‘Pesticide Use Module Applicator’ questionnaire, and 24,170 applicators returned the Phase 3 questionnaire (Fig. 1). Full text of all questionnaires is available at https://aghealth.nih.gov/collaboration/questionnaires.html.

We regularly linked cohort members to state mortality registries and the National Death Index to determine vital status. We also regularly linked participants to cancer registries in Iowa (cases reported through 2015) and North Carolina (cases reported through 2014) to determine incident cancers. Cancers were classified according to the International Classification of Diseases for Oncology, third revision (ICD-O-3) [18]. Aggressive prostate cancer was defined as having one or more of the following tumor characteristics: distant stage, poorly differentiated grade, Gleason score ≥ 7, or fatal prostate cancer (i.e. underlying cause of death was prostate cancer).

The study protocol, including implied informed consent for completion of questionnaires, was approved by all relevant institutional review boards.

### Exposure assessment

We previously published an analysis of 50 pesticides first reported on the Phase 1 enrollment questionnaire [12]. For the current analysis, we focused on those pesticides that were first reported at take-home (Phase 1 take-home questionnaire) or follow-up (Phase 2 and Phase 3 questionnaires), but not included in prior analyses (*n* = 39 pesticides). We only evaluated pesticides with 15 or more exposed aggressive PCa cases. Questions regarding pesticide use differed by questionnaire (Fig. 1). The take-home questionnaire provided a checklist of specific pesticide names/active ingredients and applicators marked those that they had ever used. The follow-up questionnaires included open-ended questions and applicators provided the name of the pesticide(s) used. In the Phase 2 questionnaire, applicators reported on use for the last year that they farmed. In the Phase 3 questionnaire, applicators were asked about use since their last questionnaire or interview (which could have been at Phase 1 enrollment, Phase 1 take-home, or Phase 2). The Phase 3 questionnaire also asked applicators to report the lifetime duration (in years) of use for each of the reported pesticides. Since the take-home and follow-up questionnaires were structured differently, this resulted in two different analytic groups: ever/never use to pesticides first reported at take-home (20 pesticides) and ever/never use to pesticides first reported at follow-up (19 pesticides). For pesticides first reported at follow-up (19 pesticides), we also evaluated lifetime duration of use (years), which was ascertained on the Phase 3 questionnaire.

Because many of these pesticides have never been evaluated in the AHS, we also assessed the plausibility of users’ duration information (excluding arsenical pesticides). We began by calculating the maximum number of years a pesticide could have been used. This was calculated by subtracting the year a pesticide/active ingredient was first registered in the U.S. from the year the individual completed the phase 3 questionnaire (2005–2010; the year participants responded to questions about duration of use for the given pesticides in the AHS). We used four different sources of information to determine registration year, including the chemical specific Registration Eligibility Decision (RED) published by the U.S. EPA, the EPA’s document on “Chemicals Registered for the First Time as Pesticidal Active Ingredients Under FIFRA” [19], different versions of the Farm Chemicals Handbooks [20] covering the timespan of the AHS, and the EXTOXNET: the Extension Toxicology Network [21], which is a Pesticide Information Project of Cooperative Extension Offices of Cornell University, Michigan State University, Oregon State University, and the University of California at Davis. We also evaluated whether any pesticide had been canceled prior to 2010 using the EPA’s chemical specific REDs, U.S. Federal Register notices, as well as the Sittig’s Handbook of Pesticides and Agricultural Chemicals [22]. We found that fluazifop-butyl and disulfoton had been canceled in the latter halves of 2004 [23] and 2009 [24], respectively. To assess plausibility of duration responses by AHS applicators, we compared the maximum number of years a pesticide could have been available for purchase to self-reported life years of use. We evaluated the frequency and proportion of applicators using a given pesticide as well as the number and proportion of users with reported years of use that were plausible.

### Analytic population

All respondents completed the enrollment questionnaire; however, responses about pesticide use from the take-home and follow-up questionnaires were structured differently resulting in two different analytic groups (Fig. 1). The first analytic group included participants that responded to the take-home questionnaire. Among the 25,291 total participants who completed the take-home questionnaire, we excluded 4368 individuals (2375 commercial applicators [did not receive the Phase 3 follow-up questionnaire], 556 women, 80 who had moved out of state, 620 with prevalent cancer or those diagnosed before completing the questionnaire, and 737 who were diagnosed with non-aggressive PCa during follow-up), leaving 20,923 private applicators (20,040 non-cases/883 aggressive PCa cases) for analysis. This analytic set was used to evaluate: (1) ever/never use as indicated on the take-home questionnaire, and (2) ever/never use considering take-home questionnaire use plus information reported at one or both follow-up questionnaire(s) (Phase 2 and/or 3). The second analytic group included participants that responded to both follow-up questionnaires (Phase 2 and Phase 3). Among the 21,142 participants that completed both follow-up questionnaires (Phase 2 and Phase 3), we excluded 2209 individuals (640 women, 70 who had moved out of state, 963 individuals with prevalent cancer or those diagnosed before completing the Phase 2 or Phase 3 questionnaire, and 536 who were diagnosed with non-aggressive PCa during follow-up), leaving 18,933 individuals (18,199 non-cases/734 aggressive PCa cases) for analysis.

### Statistical analyses

We used Cox proportional hazards models, with age as the time scale, to calculate hazard ratios (HRs) and 95% confidence intervals (CIs) for the association between individual pesticide use and aggressive PCa risk. We censored follow-up at the time of aggressive prostate cancer diagnosis, death, movement out of the state, or at December 31, 2014 for North Carolina and 2015 for Iowa, whichever came first. Supplemental Tables 1 and 2 report associations for overall PCa risk. We adjusted all models for year of birth; state (North Carolina, Iowa); family history of prostate cancer in first-degree relatives; race; and cigarette smoking status (never, former, current, missing) based on information on the Phase 1 enrollment questionnaire. Other covariates, including body mass index (BMI: underweight, normal, overweight, obese), fruit servings (< 1/day, ≥ 1/day), and leisure-time physical activity in the winter (none, > 0–2 h/week, ≥ 3 h/week), did not materially impact observed point estimates (≥ 10%) and, therefore, were not retained in models. We evaluated correlations between ever/never exposure to individual pesticides using the Pearson correlation coefficient and conducted additional adjustment for ever/never use of the three pesticides mostly highly correlated with the pesticide of interest to account for co-exposures (rho range = 0.001–0.2). We also explored adjustment of all models with pesticides previously linked to aggressive PCa in the AHS (malathion, fonofos, terbufos, and aldrin) [12] as well as with those significantly associated with PCa in the current analysis (Supplemental Table 3). However, adjustment for co-exposures did not materially impact observed point estimates and were not retained in models. We conducted exposure-response analyses for those pesticides with valid reported duration data from the Phase 3 questionnaire. We created categories for years of use based on the distribution of years reported, split at the median, among all cases. Those with reported years of use that were implausible were coded as having missing exposure in duration analyses. To compute tests for linear trend, the Wald test was used, treating the median value for each category as continuous. We evaluated potential effect modification by family history of PCa. Likelihood ratio tests were used to assess differences between strata.

Using multivariate logistic regression, we also explored possible predictors of prostate specific antigen (PSA) screening including demographic characteristics and individual pesticide use to assess whether screening could explain any observed relationships between pesticides and prostate cancer risk. Participants provided PSA screening (yes/no) information on two follow-up questionnaires (*N* = 28,880 men).

All analyses were conducted using SAS, 9.4 (SAS Institute, Cary, North Carolina) and use AHS data release P1REL201701, P2REL201701, and P3REL201701.

## Results

Of the pesticides evaluated in this analysis, acetochlor (10.8%) and picloram (10.6%) were the most commonly reported herbicides and cyfluthrin was the most commonly reported insecticide (7.6%) (Table 1). For all pesticides, most applicators reported years of use that were within the plausible range of years of market availability (average percent of applicators reporting plausible years was 97.7% [range: 88.5–100%]). Several pesticides were first registered for use at or after the initial enrollment in the AHS (*n* = 7 on or after 1993) and many others (*n* = 14) within 10-years prior to enrollment.

**Table 1:** U.S. registration year and accuracy of self-reported pesticide duration history for 38 chemicals reported on the AHS Phase 3 follow-up questionnaire (*n* = 24,170)

Common name	Class	Phase 1 Take-home questionnaire^a^	Date first registered in U.S.^b^	Applicators reported pesticide use		Users with plausible responses^c^	
				n	%	n	%
**Herbicide**							
Acetochlor	Amide		1981–1994	2604	10.8	2557	98.2
Acifluorfen	Diphenyl ether	●	1980	321	1.3	317	98.8
Bromoxynil	Nitrile	●	1965	679	2.8	678	99.9
Clethodim	Cyclohexene oxime		1992	545	2.3	523	96.0
Clomazone	Oxazole	●	1985	316	1.3	307	97.2
Clopyralid	Pyridine/Aromatic acid		1987	1262	5.2	1252	99.2
Cloransulam-methyl	Triazolopyrimidine/Amide		1997	586	2.4	554	94.5
Dimethenamid	Amide		1991	748	3.1	725	96.9
Fenoxaprop-p-ethyl	Phenoxy		1985	551	2.3	548	99.5
Fluazifop-butyl	Phenoxy	●	1983	646	2.7	638	98.8
Flumetsulam	Amide/Triazolopyrimidine		1985	1095	4.5	1087	99.3
Fomesafen	Amide/Diphenyl ether		1987	660	2.7	656	99.4
Glufosinate-ammonium	Organophosphorous		1993	1726	7.1	1685	97.6
Imazaquin	Imidazolinone	●	1986	96	0.4	94	97.9
Isoxaflutole	Oxazole/Cyclopropylisoxazole		1998	975	4.0	902	92.5
Linuron	Urea	●	1966	48	0.2	48	100.0
Maleic hydrazide	Growth inhibitor/ Gametocides		1952	387	1.6	386	99.7
Mesotrione	Benzoylcyclohexanedione		2001	2243	9.3	1985	88.5
Nicosulfuron	Urea		1990	1693	7.0	1668	98.5
Picloram	Aromatic acid/ Pyridine		1964	2563	10.6	2558	99.8
Rimsulfuron	Urea		1994	1146	4.7	1117	97.5
Sethoxydim	Cyclohexene oxime	●	1982	438	1.8	429	97.9
Simazine	Triazine	●	1957	353	1.5	353	100.0
Sodium bentazon	Unclassified	●	1974/1975–1985	382	1.6	382	100.0
Thifensulfuron-methyl	Urea	●	1989	388	1.6	369	95.1
Triclopyr	Pyridine		1979	1440	6.0	1416	98.3
**Insecticide**							
Acephate	Organophosphorous	●	1974	1197	5.0	1145	95.7
Bacillus thuringiensis	Unclassified	●	1961	200	0.8	198	99.0
Chloropicrin	Unclassified	●	1975	239	1.0	223	93.3
Cyfluthrin	Pyrethroid		1987	1849	7.6	1805	97.6
Disulfoton	Organophosphorous	●	1961	270	1.1	269	99.6
Dimethoate	Organophosphorous	●	1962	192	0.8	192	100.0
Endosulfan	Organochlorine	●	1954	203	0.8	203	100.0
Lambda-cyhalothrin	Pyrethroid		1989	876	3.6	867	99.0
Methomyl	Carbamate	●	1968	162	0.7	160	98.8
Tebupirimfos	Organophosphorous		1990–1995	1198	5.0	1148	95.8
Tefluthrin	Pyrethroid	●	1989	767	3.2	733	95.6
**Nematicide**							
1, 3-dichloropropene	Fumigant	●	1954	158	0.7	158	100.0

^a^Filled circle means chemical first appeared on take-home questionnaire; no circle means chemical first appeared on follow-up questionnaires (Phase 2/3)

^b^Range of years presented for pesticides with evidence of use prior to established registration year; for duration of market availability calculations, the lower bound of years was used (for those with a range) and rounding to a 5- or 10-year increment was calculated occurred if it fell 1 year short of this increment

^c^Plausible responses were those where total years reported were ≤ number of years calculated between Phase 3 completion year (2005–2010) and the official pesticide registration date

The distribution of major prostate cancer risk factors was similar in the two analytic populations in the current analysis as well as compared to the full cohort of private applicators (Supplemental Table 4). The total number of PCa cases was 3169, with aggressive cases making up 54.6% of all cases.

For pesticides first reported on the take-home questionnaire, there was a significantly increased risk of aggressive PCa among ever users of dimethoate (HR =1.37, 95% CI = 1.04, 1.80) compared to never users (Table 2). When we included additional exposure information reported in follow-up questionnaires, the risk for dimethoate was similar (HR =1.29, 95% CI = 0.98, 1.70). The increased risk for dimethoate was unchanged with additional adjustment for highly correlated pesticides or with any other pesticides linked to PCa (data not shown). The association between dimethoate use and aggressive PCa appeared stronger in those with a positive family history of PCa (HR = 1.84, 95% CI = 0.99, 3.43) compared to those without a family history of PCa (HR = 1.31, 95% CI = 0.96, 1.79), but the interaction was not statistically significant (P_interaction_ = 0.32; data not shown). We observed slightly elevated HRs between ever use of bromoxynil (HR =1.17, 95% CI = 0.99, 1.37), linuron (HR =1.19, 95% CI = 0.99, 1.42), and sethoxydim (HR =1.12, 95% CI = 0.96, 1.30) and aggressive PCa, but none were statistically significant (Table 2). We did not observe any statistically significant interactions between any specific pesticide use reported on the take-home questionnaire and family history of prostate cancer.

**Table 2:** Association between pesticide (ever/never use) and aggressive PCa for those pesticides first reported at the Phase 1 take-home (TH) questionnaire in the Agricultural Health Study (AHS)

Common name	Phase 1 take-home questionnaire only (*n* = 20,923)			Phase 1 take-home questionnaire including follow-up (Phase 2/Phase 3 questionnaire) exposure information^b^		
	Non-case	Aggressive PCa	HR^a^ (95% CI)	Non-case	Aggressive PCa	HR^a^ (95% CI)
**Herbicide**						
Acifluorfen						
Never use	16,626	744	1	13,371	621	1
Ever use	3414	139	1.08 (0.90, 1.30)	3642	146	1.06 (0.89, 1.27)
Bromoxynil						
Never use	14,609	628	1	11,557	523	1
Ever use	5431	255	1.17 (0.99, 1.37)	5758	266	1.14 (0.96, 1.34)
Clomazone						
Never use	16,850	756	1	13,471	632	1
Ever use	3190	127	0.96 (0.79, 1.17)	3462	132	0.94 (0.78, 1.14)
Fluazifop-butyl						
Never use	16,682	750	1	13,077	620	1
Ever use	3358	133	1.07 (0.89, 1.29)	3909	149	1.01 (0.84, 1.21)
Imazaquin						
Never use	17,052	770	1	13,880	646	1
Ever use	2988	113	0.99 (0.81, 1.20)	3101	116	0.99 (0.81, 1.20)
Linuron						
Never use	17,509	742	1	14,267	622	1
Ever use	2531	141	1.19 (0.99, 1.42)	2548	143	1.19 (0.996, 1.43)
Sethoxydim						
Never use	14,404	647	1	11,486	546	1
Ever use	5636	236	1.12 (0.96, 1.30)	5835	239	1.07 (0.91, 1.24)
Simazine						
Never use	18,454	803	1	14,922	671	1
Ever use	1586	80	1.19 (0.94, 1.51)	1724	86	1.17 (0.93, 1.48)
Sodium bentazon						
Never use	12,911	573	1	10,250	476	1
Ever use	7129	310	1.08 (0.93, 1.25)	7332	317	1.06 (0.91, 1.23)
Thifensulfuron-methyl						
Never use	18,108	821	1	14,355	668	1
Ever use	1932	62	0.91 (0.70, 1.18)	2389	83	1.00 (0.79, 1.26)
**Insecticide**						
Acephate						
Never use	18,069	811	1	14,377	671	1
Ever use	1971	72	1.06 (0.82, 1.39)	2427	85	1.00 (0.77, 1.29)
Bacillus thuringiensis						
Never use	18,256	813	1	14,855	689	1
Ever use	1784	70	1.00 (0.77, 1.29)	1904	71	0.93 (0.72, 1.20)
Chloropicrin						
Never use	19,644	865	1	15,944	730	1
Ever use	396	18	1.48 (0.92, 2.38)	566	20	1.21 (0.77, 1.91)
Disulfoton						
Never use	18,332	815	1	14,927	684	1
Ever use	1708	68	0.96 (0.74, 1.26)	1807	72	0.96 (0.74, 1.25)
Dimethoate						
Never use	19,262	829	1	15,673	695	1
Ever use	778	54	**1.37 (1.04, 1.80)**	870	55	1.29 (0.98, 1.70)
Endosulfan						
Never use	19,407	860	1	15,777	722	1
Ever use	633	23	0.85 (0.56, 1.30)	750	25	0.78 (0.51, 1.17)
Methomyl						
Never use	18,532	827	1	15,133	698	1
Ever use	1508	56	1.05 (0.78, 1.40)	1597	58	1.03 (0.77, 1.37)
Tefluthrin						
Never use	18,635	828	1	14,805	684	1
Ever use	1405	55	0.99 (0.75, 1.31)	1855	71	0.98 (0.76, 1.25)
**Nematicide**						
1, 3-dichloropropene						
Never use	19,172	848	1	15,669	716	1
Ever use	868	35	1.14 (0.80, 1.62)	944	37	1.11 (0.78, 1.57)
**Other**						
Arsenical pesticides^c^						
Never use	19,309	828	1	–	–	–
Ever use	731	55	1.17 (0.88, 1.55)	–	–	–

^a^Using age as the time metric and adjusted for state, birth year, family history of PCa, race, and smoking status

^b^Numbers add to less than total number of take-home responders (*n* = 20,923) due to missing responses for follow-up use information

^c^Arsenical pesticides consist of lead arsenate (insecticide), organic arsenic (herbicide), and inorganic arsenic (herbicide); only reported use reported on take-home questionnaire

For pesticides first reported at Phase 2 and/or Phase 3 follow-ups, there was a significant inverse association between ever use of triclopyr and aggressive PCa (HR =0.68, 95% CI = 0.48, 0.95) (Table 3). The risk was not impacted by additional adjustment for other highly correlated pesticides or with any other pesticide linked to PCa (data not shown). Exposure-response analyses evaluating years of use of triclopyr showed that the HR was lowest among those who reported using triclopyr ≥ 4 years compared to never users (HR =0.44, 95% CI = 0.26, 0.77, *n* = 13 exposed cases, *p*-value for trend = 0.004, Table 4). We observed no other positive or inverse associations between ever use or duration of use of other pesticides first reported at follow-up and aggressive PCa (Tables 3 and 4).

**Table 3:** Association between pesticide (ever/never use) and aggressive PCa for those pesticides first reported at follow-up (Phase 2 and Phase 3 questionnaires) in the Agricultural Health Study (AHS)

Common name	Follow-up questionnaire (Phase 2 and Phase 3)^b^ *N* = 18,933		
	Non-case	Aggressive PCa	HR^a^ (95% CI)
**Herbicide**			
Acetochlor			
Never use	15,110	640	1
Ever use	3089	94	0.87 (0.70, 1.09)
Clethodim			
Never use	17,526	717	1
Ever use	673	17	1.03 (0.64, 1.68)
Clopyralid			
Never use	16,513	690	1
Ever use	1686	44	0.79 (0.58, 1.08)
Cloransulam-methyl			
Never use	17,453	709	1
Ever use	746	25	1.13 (0.76, 1.69)
Dimethenamid			
Never use	17,238	709	1
Ever use	961	25	0.82 (0.55, 1.23)
Fenoxaprop-p-ethyl			
Never use	17,228	703	1
Ever use	971	31	1.00 (0.69, 1.44)
Flumetsulam			
Never use	16,612	687	1
Ever use	1587	47	0.91 (0.67, 1.23)
Fomesafen			
Never use	16,955	690	1
Ever use	1244	44	1.08 (0.80, 1.48)
Glufosinate-ammonium			
Never use	16,538	692	1
Ever use	1661	42	0.81 (0.59, 1.11)
Isoxaflutole			
Never use	17,172	703	1
Ever use	1027	31	1.03 (0.72, 1.49)
Maleic hydrazide			
Never use	17,326	709	1
Ever use	873	25	0.91 (0.60, 1.38)
Mesotrione			
Never use	16,312	692	1
Ever use	1887	42	0.80 (0.58, 1.10)
Nicosulfuron			
Never use	15,931	674	1
Ever use	2268	60	0.78 (0.60, 1.03)
Picloram			
Never use	15,848	652	1
Ever use	2351	82	0.97 (0.77, 1.23)
Rimsulfuron			
Never use	16,728	694	1
Ever use	1471	40	0.83 (0.60, 1.15)
Triclopyr			
Never use	16,788	699	1
Ever use	1411	35	**0.68 (0.48, 0.95)**
**Insecticide**			
Cyfluthrin			
Never use	16,399	691	1
Ever use	1800	43	0.81 (0.59, 1.10)
Lambda-cyhalothrin			
Never use	17,257	712	1
Ever use	942	22	0.96 (0.63, 1.47)
Tebupirimfos			
Never use	17,036	702	1
Ever use	1163	32	0.86 (0.60, 1.23)

^a^Using age as the time metric and adjusted for state, birth year, family history of PCa, race, and smoking status

^b^Numbers may not add up to total, due to missing responses for pesticides

**Table 4:** Association between duration of pesticide use (in years) and aggressive PCa for those pesticides first reported at follow-up (Phase 2 and Phase 3 questionnaires) in the Agricultural Health Study (AHS)^b^

Common name	Non-case	Aggressive PCa	HR (95% CI)^a^
**Herbicide**			
Acetochlor			
Never use	15,097	633	1
< 9 years	1213	28	0.76 (0.52, 1.12)
≥ 9 years	882	30	1.12 (0.77, 1.63)
p-trend	0.96		
Clopyralid			
Never use	16,504	684	1
< 5 years	469	9	0.66 (0.34, 1.28)
≥ 5 years	565	15	0.88 (0.53, 1.47)
p-trend	0.39		
Glufosinate-ammonium			
Never use	16,516	683	1
< 5 years	613	12	0.67 (0.38, 1.20)
≥ 5 years	526	16	1.03 (0.62, 1.70)
p-trend	0.79		
Mesotrione			
Never use	16,284	680	1
< 4 years	753	17	0.82 (0.50, 1.33)
≥ 4 years	856	17	0.77 (0.48, 1.26)
p-trend	0.22		
Nicosulfuron			
Never use	15,921	668	1
< 5 years	560	15	0.88 (0.53, 1.48)
≥ 5 years	820	21	0.84 (0.54, 1.30)
p-trend	0.39		
Picloram			
Never use	15,817	637	1
< 6 years	899	32	1.15 (0.80, 1.65)
≥ 6 years	1145	32	0.79 (0.55, 1.14)
p-trend	0.28		
Rimsulfuron			
Never use	16,717	690	1
< 4 years	333	10	1.00 (0.54, 1.88)
≥ 4 years	573	14	0.78 (0.46, 1.32)
p-trend	0.39		
Triclopyr			
Never use	16,770	690	1
< 4 years	282	10	0.99 (0.53, 1.85)
≥ 4 years	848	13	**0.44 (0.26, 0.77)**
p-trend	**0.004**		
**Insecticide**			
Cyfluthrin			
Never use	16,380	686	1
< 6 years	803	17	0.71 (0.44, 1.15)
≥ 6 years	645	14	0.82 (0.48, 1.39)
p-trend	0.22		
Tebupirimfos			
Never use	17,022	698	1
< 6 years	540	12	0.69 (0.39, 1.23)
≥ 6 years	381	12	1.09 (0.61, 1.93)
p-trend	0.67		

^a^Using age as the time metric and adjusted for state, birth year, family history of PCa, race, and smoking status

^b^Numbers add to less than total number of follow-up responders (*n* = 18,933) due to missing responses for duration of use information

Supplemental Table 5 shows various predictors of PSA screening. Eighty-one percent of all men (23,416/28,880) and 87% of men over the age of 50 (22,217/25,421) had received a PSA test (Supplemental Table 5). Nearly all PCa cases were screened for PSA (overall PCa: 92.6%, aggressive PCa: 91.7%;). State, family history, age, marriage status, smoking status, educational attainment, and BMI were all predictors of PSA. Neither dimethoate nor triclopyr were associated with PSA testing, after controlling for other predictors.

## Discussion

This study reports on the risk of aggressive PCa in relation to use of 39 individual pesticides that have not been previously evaluated in a large prospective cohort of private pesticide applicators. We found no association between the use of most of these pesticides and risk of aggressive prostate cancer. However, we did find a significantly elevated risk of aggressive PCa among ever users of the OP insecticide dimethoate and an inverse association between ever use of the herbicide triclopyr. We also found suggestive associations with commonly used herbicides such as bromoxynil, linuron, and sethoxydim and aggressive PCa.

Dimethoate (O,O-dimethyl S-methylcarbamoylmethyl phosphorodithioate) is a dithioate OP insecticide. It was first registered for use in the U.S. in 1962 for use on alfalfa, wheat, cotton, and corn [25]. In 1983, the International Agency for Research on Cancer (IARC) failed to classify dimethoate regarding human carcinogenicity due to lack of available data [26]. In 1991, the U.S. EPA classified dimethoate as a possible human carcinogen (Group C). Dimethoate exhibited carcinogenic effects in some strains of rats and mice, with neoplasms occurring in the endocrine organs, liver, and lymphatic systems [27]; however, there have been no links to prostate cancer in humans. In this analysis, we observed a higher risk for aggressive PCa among applicators who reported dimethoate use. Pesticide applicators who reported use of dimethoate were more likely to report application methods related to animals, which is also consistent with known uses for dimethoate [28]. Our inclusion of information on pesticide use during follow-up yielded a similar magnitude of elevated risk, although the association was no longer statistically significant. This may be due to a decrease in sample size from non-response in the follow-up questionnaires. Exposure-response analyses for duration of dimethoate use were also precluded due to a lack of use during follow-up for this insecticide (primarily historical use). Work practice information from pesticide applicators who used dimethoate suggested that variations in application methods and use of personal protective equipment did not materially change the observed association between dimethoate and aggressive prostate cancer (data not shown).

To our knowledge, no previous epidemiologic studies have reported on the specific relationship between dimethoate and PCa (or any other cancer site); however, some studies found associations between aggressive PCa risk and exposure to other dithioate insecticides, including fonofos, malathion, terbufos, and azinphos-methyl [12, 29]. The mechanism of pesticidal action of OP insecticides is to inhibit the enzyme that breaks down the neurotransmitter acetylcholine; however, this mechanism has not been explicitly linked to prostate cancer risk. Recently, a large genomic analysis of prostate cancer in 140,000 men found that genetic variants in pathways related to neurotransmission release were enriched in prostate cancer [30]. We also have suggested that pesticides may interact with genetic variants in signal transduction and cellular communication pathways affected by neurotransmission [31]. Thus, a link between acetylcholinesterase inhibition and prostate cancer is plausible. These findings indicate the need for future work on OP insecticides (specifically organodithioate insecticides) to identify a biologically plausible link to prostate cancer.

We also found an inverse association between triclopyr use and aggressive PCa. Exposure-response analyses, using lifetime years of use among those who completed the Phase 3 questionnaire showed that increasing years of use was significantly associated with a decreased risk of prostate cancer (p-trend = 0.008). In 1995, the U.S. EPA deemed triclopyr not classifiable as to human carcinogenicity (Group D) because no epidemiologic studies had been conducted and only suggestive results had been seen in animal studies [32]. Triclopyr (3,5,6-trichloro-2-pyridyloxyacetic acid) is a pyridine herbicide, which breaks down into 3,5,6-trichloro-2-pyridinol (TCPy) in the soil. Studies have suggested that TCPy can persist for a maximum of 9 months, but most soil samples exhibited a half-life of less than 90 days [33]. There have been some epidemiologic studies evaluating the association between the TCPy—also a metabolite of the insecticide chlorpyrifos—and sex and thyroid hormones [34–36]. These studies observed decreased levels of testosterone [34], estradiol [33], and altered levels of thyroid function markers, several of which have been suspected in the etiology of prostate cancer [37–43]. However, in humans, triclopyr is rapidly eliminated with more than 80% unchanged when excreted in urine, thus, it is unclear if there would be hormonal effects in humans from TCPy exposure [44]. There are no direct data about possible endocrine disrupting properties of triclopyr and alternative explanations including the possibility of a chance finding cannot be ruled out. This pesticide, however, is commonly mixed with suspected endocrine disrupting chlorophenoxy herbicides [45, 46] because of a common mechanism of action (triclopyr is the pyridine analogue of 2,4,5- trichlorophenoxyacetic acid).

There were small increased risks of aggressive PCa from reported use of the herbicides bromoxynil, linuron, and sethoxydim, but none were statistically significant. Bromoxynil, or bromoxynil phenol, was classified by the U.S. EPA as a possible human carcinogen (Group C) in 1998 based on only a few studies conducted in animals [47]. To our knowledge, there have been no human studies published on the health effects of bromoxynil. Linuron, or 3-(3,4-dichlorophenyl)-1-methoxy-1-methylurea, was classified by the U.S. EPA as a possible human carcinogen (Group C) in 1990 due to observed testicular effects in rats, including interstitial cell hyperplasia and adenomas [48]. In rats, linuron is considered to play a toxic role in the male reproductive system as it has been indicated to significantly alter the expression of genes associated with testosterone synthesis, cell proliferation, and apoptosis [49]. Sethoxydim, or 2[1-(ethoxyimino)butyl]-5-[2-(ethylthio)propyl]-3-hydroxy-2-cyclohexen-1-one, was classified by the U.S. EPA in 2005 as not likely to be carcinogenic in humans based on the lack of evidence of carcinogenicity in rats and mice [50]. More data in humans are needed to adequately understand the relationship between exposure to these chemicals and cancer risk.

A strength of our study is the large size and the detailed information on pesticide use that allows for an assessment of individual pesticides in relation to the development of aggressive PCa. Pesticide applicators can reliably report on their use of pesticides [51, 52]. We found the reported duration of pesticide use by applicators showed a high level of agreement with the duration of plausible market availability across all pesticides. In addition, we focused our analyses to clinically relevant subtypes of PCa to identify risk factors for this more aggressive subtype of disease. When we evaluated the possible influence of PSA screening on risk estimates, we found that use of individual pesticides associated with risk was not related to a history of PSA screening (eliminating potential biases due to screening).

There were also limitations. The ability to evaluate cumulative lifetime use for some pesticides first reported in Phase 2 and Phase 3 is limited which may result in non-differential exposure misclassification. However, this exposure misclassification would likely result in biasing our results toward the null [53]. Moreover, this is not an issue for the pesticides reported in Table 2, where lifetime use before enrollment was assessed. We were also not able to conduct exposure-response analyses for all users of a given pesticide because lifetime duration of use information was only ascertained during the Phase 3 questionnaire. And in some instances, duration of use for pesticides that were reported had small numbers for adequately powered exposure-response analysis. For many of the pesticides reported in Table 3, registration dates are relatively close to the time of prostate cancer diagnosis (approximately 5–15 years). Although there are limited data on the latent period for prostate cancer, it is possible that insufficient time has lapsed between exposure and cancer development for many of the pesticides evaluated here. In fact, thus far, positive associations for prostate cancer have only been observed for insecticides that were first registered for use primarily in the 1940s–1960s, suggesting a long latent period for pesticide-induced prostate cancer. Finally, we also made several comparisons, increasing the likelihood of a chance finding.

## Conclusion

We found a significantly elevated risk of aggressive PCa among ever users of the OP insecticide dimethoate and an inverse association between ever use of the herbicide triclopyr. The association we observed between dimethoate and aggressive PCa adds another dithioate insecticide to the list of OPs associated with PCa risk in the AHS. Few other pesticides were associated with aggressive PCa, but extended follow-up of the cohort is warranted for this long latency cancer.

## Supplementary information

**Additional file 1: Table S1.** Association between pesticide (ever/never use) and overall PCa for those pesticides first reported at the Phase 1 take-home (TH) questionnaire in the Agricultural Health Study (AHS).

**Additional file 2: Table S2.** Association between pesticide (ever/never use) and overall PCa for those pesticides first reported at follow-up (Phase 2 and Phase 3 questionnaires) in the Agricultural Health Study (AHS).

**Additional file 3: Table S3.** Correlation analysis between take-home pesticides and organophosphates previously identified as associated with aggressive PCa in the AHS.

**Additional file 4: Table S4.** Selected characteristics of participants in the Agricultural Health Study (AHS) cohort; aggressive prostate cancer (PCa) cases reported through 2014 in North Carolina and 2015 in Iowa^a^.

**Additional file 5: Table S5.** Prostate specific antigen (PSA) frequencies by selected characteristics^a^.

## Data Availability

The Agricultural Health Study has procedures in place for access of study data that can be found at (https://aghealth.nih.gov/collaboration/process.html).

## Abbreviations

AHS: Agricultural Health Study
BMI: Body mass index
CATI: Computer assisted telephone interview
CI: Confidence interval
EPA: Environmental Protection Agency
HR: Hazard ratio
IARC: International Agency for Research on Cancer
ICD-O-3: International Classification of Diseases for Oncology, third revision
OC: Organochlorine
OP: Organophosphate
PCa: Prostate cancer
PSA: Prostate specific antigen
RED: Registration Eligibility Decision
