# Supplementary material for: Pesticide exposure and risk of aggressive prostate cancer among private pesticide applicators
Source: Environ Health. 2020 Mar 5;19:30. doi: 10.1186/s12940-020-00583-0 (PMC7059337; doi:10.1186/s12940-020-00583-0)
Supplement: Supplementary file 1 — Additional file 1: Table S1. Association between pesticide (ever/never use) and overall PCa for those pesticides first reported at the Phase 1 take-home (TH) questionnaire in the Agricultural Health Study (AHS). [file 12940_2020_583_MOESM1_ESM.docx]

Supplemental Table 1. Association between pesticide (ever/never use) and overall PCa for those pesticides first reported at the Phase 1 take-home (TH) questionnaire in the Agricultural Health Study (AHS)

| **Common name** | | Phase 1 take-home questionnaire only  (n = 21,660) | | | Phase 1 take-home questionnaire including follow-up (Phase 2/Phase 3 questionnaire) exposure information^b^ | | |
| --- | --- | --- | --- | --- | --- | --- | --- |
|  |  | Non-case | Overall PCa | HR^a^  (95% CI) | Non-case | Overall PCa | HR^a^  (95% CI) |
| **Herbicide** | |  |  |  |  |  |  |
|  | Acifluorfen |  |  |  |  |  |  |
|  | Never use | 16,626 | 1,381 | 1 | 13,371 | 1,152 | 1 |
|  | Ever use | 3,414 | 239 | 1.00 (0.87, 1.14) | 3,642 | 251 | 0.98 (0.85, 1.12) |
|  | Bromoxynil |  |  |  |  |  |  |
|  | Never use | 14,609 | 1,185 | 1 | 11,557 | 979 | 1 |
|  | Ever use | 5,431 | 435 | 1.13 (0.999, 1.28) | 5,758 | 456 | 1.11 (0.98, 1.26) |
|  | Clomazone |  |  |  |  |  |  |
|  | Never use | 16,850 | 1,382 | 1 | 13,471 | 1,152 | 1 |
|  | Ever use | 3,190 | 238 | 1.04 (0.90, 1.20) | 3,462 | 253 | 1.04 (0.90, 1.19) |
|  | Fluazifop-butyl |  |  |  |  |  |  |
|  | Never use | 16,682 | 1,369 | 1 | 13,077 | 1,119 | 1 |
|  | Ever use | 3,358 | 251 | 1.13 (0.99, 1.29) | 3,909 | 285 | 1.10 (0.96, 1.25) |
|  | Imazaquin |  |  |  |  |  |  |
|  | Never use | 17,052 | 1,417 | 1 | 13,880 | 1,187 | 1 |
|  | Ever use | 2,988 | 203 | 0.99 (0.85, 1.15) | 3,101 | 206 | 0.98 (0.84, 1.13) |
|  | Linuron |  |  |  |  |  |  |
|  | Never use | 17,509 | 1,373 | 1 | 14,267 | 1,149 | 1 |
|  | Ever use | 2,531 | 247 | 1.12 (0.98, 1.28) | 2,548 | 250 | 1.12 (0.98, 1.29) |
|  | Sethoxydim |  |  |  |  |  |  |
|  | Never use | 14,404 | 1,199 | 1 | 11,486 | 1,001 | 1 |
|  | Ever use | 5,636 | 421 | 1.09 (0.97, 1.21) | 5,835 | 430 | 1.06 (0.94, 1.18) |
|  | Simazine |  |  |  |  |  |  |
|  | Never use | 18,454 | 1,479 | 1 | 14,922 | 1,228 | 1 |
|  | Ever use | 1,586 | 141 | 1.08 (0.90, 1.29) | 1,724 | 150 | 1.05 (0.88, 1.25) |
|  | Sodium bentazon |  |  |  |  |  |  |
|  | Never use | 12,911 | 1,055 | 1 | 10,250 | 872 | 1 |
|  | Ever use | 7,129 | 565 | 1.12 (1.01, 1.25) | 7,332 | 575 | 1.10 (0.98, 1.23) |
|  | Thifensulfuron-methyl |  |  |  |  |  |  |
|  | Never use | 18,108 | 1,501 | 1 | 14,355 | 1,224 | 1 |
|  | Ever use | 1,932 | 119 | 0.99 (0.82, 1.19) | 2,389 | 157 | 1.06 (0.90, 1.26) |
| **Insecticide** | |  |  |  |  |  |  |
|  | Acephate |  |  |  |  |  |  |
|  | Never use | 18,069 | 1,479 | 1 | 14,377 | 1,218 | 1 |
|  | Ever use | 1,971 | 141 | 1.03 (0.85, 1.24) | 2,427 | 167 | 0.97 (0.81, 1.16) |
|  | Bacillus thuringiensis |  |  |  |  |  |  |
|  | Never use | 18,256 | 1,468 | 1 | 14,855 | 1,234 | 1 |
|  | Ever use | 1,784 | 152 | 1.11 (0.93, 1.33) | 1,904 | 157 | 1.06 (0.89, 1.27) |
|  | Chloropicrin |  |  |  |  |  |  |
|  | Never use | 19,644 | 1,590 | 1 | 15,944 | 1,333 | 1 |
|  | Ever use | 396 | 30 | 1.27 (0.88, 1.84) | 566 | 37 | 1.16 (0.83, 1.62) |
|  | Disulfoton |  |  |  |  |  |  |
|  | Never use | 18,332 | 1,478 | 1 | 14,927 | 1,233 | 1 |
|  | Ever use | 1,708 | 142 | 1.02 (0.85, 1.23) | 1,807 | 152 | 1.03 (0.86, 1.24) |
|  | Dimethoate |  |  |  |  |  |  |
|  | Never use | 19,262 | 1,541 | 1 | 15,673 | 1,289 | 1 |
|  | Ever use | 778 | 79 | 1.09 (0.87, 1.37) | 870 | 81 | 1.03 (0.83, 1.30) |
|  | Endosulfan |  |  |  |  |  |  |
|  | Never use | 19,407 | 1,566 | 1 | 15,777 | 1,311 | 1 |
|  | Ever use | 633 | 54 | 1.02 (0.77, 1.34) | 750 | 57 | 0.90 (0.69, 1.19) |
|  | Methomyl |  |  |  |  |  |  |
|  | Never use | 18,532 | 1,506 | 1 | 15,133 | 1,269 | 1 |
|  | Ever use | 1,508 | 114 | 1.05 (0.86, 1.29) | 1,597 | 118 | 1.03 (0.84, 1.26) |
|  | Tefluthrin |  |  |  |  |  |  |
|  | Never use | 18,635 | 1,518 | 1 | 14,805 | 1,249 | 1 |
|  | Ever use | 1,405 | 102 | 1.06 (0.87, 1.30) | 1,855 | 133 | 1.06 (0.88, 1.27) |
| **Nematicide** | |  |  |  |  |  |  |
|  | 1, 3-dichloropropene |  |  |  |  |  |  |
|  | Never use | 19,172 | 1,554 | 1 | 15,669 | 1,308 | 1 |
|  | Ever use | 868 | 66 | 1.07 (0.83, 1.38) | 944 | 71 | 1.06 (0.83, 1.36) |
| **Other** | |  |  |  |  |  |  |
|  | Arsenical pesticides^c^ |  |  |  |  |  |  |
|  | Never use | 19,309 | 1,519 | 1 | -- | -- | -- |
|  | Ever use | 731 | 101 | 1.10 (0.90, 1.36) | -- | -- | -- |

^a^ Using age as the time metric and adjusted for state, birth year, family history of PCa, race, and smoking status

^b^ Numbers add to less than total number of take-home responders (n=20,923) due to missing responses for follow-up use information

^c^ Arsenical pesticides consist of lead arsenate (insecticide), organic arsenic (herbicide), and inorganic arsenic (herbicide); only reported use reported on take-home questionnaire
