# Supplementary material for: Pesticide exposure and risk of aggressive prostate cancer among private pesticide applicators
Source: Environ Health. 2020 Mar 5;19:30. doi: 10.1186/s12940-020-00583-0 (PMC7059337; doi:10.1186/s12940-020-00583-0)
Supplement: Supplementary file 2 — Additional file 2: Table S2. Association between pesticide (ever/never use) and overall PCa for those pesticides first reported at follow-up (Phase 2 and Phase 3 questionnaires) in the Agricultural Health Study (AHS). [file 12940_2020_583_MOESM2_ESM.docx]

Supplemental Table 2. Association between pesticide (ever/never use) and overall PCa for those pesticides first reported at follow-up (Phase 2 and Phase 3 questionnaires) in the Agricultural Health Study (AHS)

| **Common name** | | Follow-up questionnaire (Phase 2 and Phase 3)^b^  N=19,469 | | |
| --- | --- | --- | --- | --- |
|  |  | Non-case | Overall PCa | HR^a^  (95% CI) |
| **Herbicide** | |  |  |  |
|  | Acetochlor |  |  |  |
|  | Never use | 15,110 | 1,112 | 1 |
|  | Ever use | 3,089 | 158 | 0.87 (0.73, 1.04) |
|  | Clethodim |  |  |  |
|  | Never use | 17,526 | 1,240 | 1 |
|  | Ever use | 673 | 30 | 1.05 (0.73, 1.51) |
|  | Clopyralid |  |  |  |
|  | Never use | 16,513 | 1,184 | 1 |
|  | Ever use | 1,686 | 86 | 0.93 (0.74, 1.16) |
|  | Cloransulam-methyl |  |  |  |
|  | Never use | 17,453 | 1,225 | 1 |
|  | Ever use | 746 | 45 | 1.19 (0.88, 1.61) |
|  | Dimethenamid |  |  |  |
|  | Never use | 17,238 | 1,224 | 1 |
|  | Ever use | 961 | 46 | 0.90 (0.67, 1.21) |
|  | Fenoxaprop-p-ethyl |  |  |  |
|  | Never use | 17,228 | 1,207 | 1 |
|  | Ever use | 971 | 63 | 1.21 (0.94, 1.57) |
|  | Flumetsulam |  |  |  |
|  | Never use | 16,612 | 1,181 | 1 |
|  | Ever use | 1,587 | 89 | 1.03 (0.83, 1.29) |
|  | Fomesafen |  |  |  |
|  | Never use | 16,955 | 1,188 | 1 |
|  | Ever use | 1,244 | 82 | 1.20 (0.96, 1.51) |
|  | Glufosinate-ammonium |  |  |  |
|  | Never use | 16,538 | 1,203 | 1 |
|  | Ever use | 1,661 | 67 | **0.75 (0.59, 0.97)** |
|  | Isoxaflutole |  |  |  |
|  | Never use | 17,172 | 1,222 | 1 |
|  | Ever use | 1,027 | 48 | 0.94 (0.70, 1.26) |
|  | Maleic hydrazide |  |  |  |
|  | Never use | 17,326 | 1,215 | 1 |
|  | Ever use | 873 | 55 | 1.11 (0.83, 1.47) |
|  | Mesotrione |  |  |  |
|  | Never use | 16,312 | 1,205 | 1 |
|  | Ever use | 1,887 | 65 | **0.72 (0.56, 0.93)** |
|  | Nicosulfuron |  |  |  |
|  | Never use | 15,931 | 1,153 | 1 |
|  | Ever use | 2,268 | 117 | 0.92 (0.76, 1.12) |
|  | Picloram |  |  |  |
|  | Never use | 15,848 | 1,151 | 1 |
|  | Ever use | 2,351 | 119 | 0.83 (0.68, 1.00) |
|  | Rimsulfuron |  |  |  |
|  | Never use | 16,728 | 1,190 | 1 |
|  | Ever use | 1,471 | 80 | 1.00 (0.79, 1.26) |
|  | Triclopyr |  |  |  |
|  | Never use | 16,788 | 1,208 | 1 |
|  | Ever use | 1,411 | 62 | **0.69 (0.54, 0.90)** |
| **Insecticide** | |  |  |  |
|  | Cyfluthrin |  |  |  |
|  | Never use | 16,399 | 1,189 | 1 |
|  | Ever use | 1,800 | 81 | 0.89 (0.71, 1.12) |
|  | Lambda-cyhalothrin |  |  |  |
|  | Never use | 17,257 | 1,239 | 1 |
|  | Ever use | 942 | 31 | 0.78 (0.54, 1.11) |
|  | Tebupirimfos |  |  |  |
|  | Never use | 17,036 | 1,213 | 1 |
|  | Ever use | 1,163 | 57 | 0.90 (0.69, 1.18) |

^a^ Using age as the time metric and adjusted for state, birth year, family history of PCa, race, and smoking status

^b^ Numbers may not add up to total, due to missing responses for pesticides
