# Supplementary material for: Pesticide exposure and risk of aggressive prostate cancer among private pesticide applicators
Source: Environ Health. 2020 Mar 5;19:30. doi: 10.1186/s12940-020-00583-0 (PMC7059337; doi:10.1186/s12940-020-00583-0)
Supplement: Supplementary file 3 — Additional file 3: Table S3. Correlation analysis between take-home pesticides and organophosphates previously identified as associated with aggressive PCa in the AHS. [file 12940_2020_583_MOESM3_ESM.docx]

Supplemental Table 3. Correlation analysis between take-home pesticides and organophosphates previously identified as associated with aggressive PCa in the AHS

|  | **Bromoxynil** | **Linuron** | **Sethoxydim** | **Dimethoate** | **Fonofos** | **Terbufos** | **Malathion** | **Aldrin** |
| --- | --- | --- | --- | --- | --- | --- | --- | --- |
| **Bromoxynil** | 1.00 | 0.07 | 0.26 | 0.06 | 0.14 | 0.16 | 0.11 | 0.13 |
| **Linuron** | 0.07 | 1.00 | 0.29 | 0.09 | 0.06 | 0.10 | 0.10 | 0.16 |
| **Sethoxydim** | 0.26 | 0.29 | 1.00 | 0.13 | 0.05 | 0.09 | 0.14 | 0.08 |
| **Dimethoate** | 0.06 | 0.09 | 0.13 | 1.00 | 0.02 | 0.02 | 0.08 | 0.09 |
| **Fonofos** | 0.14 | 0.06 | 0.05 | 0.02 | 1.00 | 0.29 | 0.14 | 0.19 |
| **Terbufos** | 0.16 | 0.10 | 0.09 | 0.02 | 0.29 | 1.00 | 0.18 | 0.20 |
| **Malathion** | 0.11 | 0.10 | 0.14 | 0.08 | 0.14 | 0.18 | 1.00 | 0.22 |
| **Aldrin** | 0.13 | 0.16 | 0.08 | 0.09 | 0.19 | 0.20 | 0.22 | 1.00 |
