# Supplementary material for: Pesticide exposure and risk of aggressive prostate cancer among private pesticide applicators
Source: Environ Health. 2020 Mar 5;19:30. doi: 10.1186/s12940-020-00583-0 (PMC7059337; doi:10.1186/s12940-020-00583-0)
Supplement: Supplementary file 4 — Additional file 4: Table S4. Selected characteristics of participants in the Agricultural Health Study (AHS) cohort; aggressive prostate cancer (PCa) cases reported through 2014 in North Carolina and 2015 in Iowaa. [file 12940_2020_583_MOESM4_ESM.docx]

Supplemental Table 4. Selected characteristics of participants in the Agricultural Health Study (AHS) cohort; aggressive prostate cancer (PCa) cases reported through 2014 in North Carolina and 2015 in Iowa.^a^

| Selected Characteristics | | Full cohort^b^ | | | | | | Responders to Phase 1 take-home (TH) questionnaire^c^ | | | | Responders to both follow-up questionnaires (Phase 2 and Phase 3)^d^ | | | |
| --- | --- | --- | --- | --- | --- | --- | --- | --- | --- | --- | --- | --- | --- | --- | --- |
|  |  | N=49,829 | | | | | | N = 20,923 | | | | N = 18,933 | | | |
|  |  | Non-case | | Overall PCa | | Aggressive PCa | | Non-case | | Aggressive PCa | | Non-case | | Aggressive PCa | |
|  |  | N | % | N | % | N | % | N | % | N | % | N | % | N | % |
| All subjects | | 46,660 | 100 | 3,169 | 100 | 1,730 | 100 | 20,040 | 100 | 883 | 100 | 18,199 | 100 | 734 | 100 |
| Attained age (years) | |  |  |  |  |  |  |  |  |  |  |  |  |  |  |
|  | < 40 | 711 | 1.5 | 2 | 0.1 | 1 | 0.1 | 192 | 1.0 | 0 | 0.0 | 85 | 0.5 | 0 | 0.0 |
|  | 40 – 49 | 4,323 | 9.3 | 45 | 1.4 | 19 | 1.1 | 1,445 | 7.2 | 5 | 0.6 | 1,058 | 5.8 | 7 | 1.0 |
|  | 50 – 59 | 12,267 | 26.3 | 571 | 18.0 | 287 | 16.6 | 4,470 | 22.3 | 115 | 13.0 | 4,297 | 23.6 | 105 | 14.3 |
|  | 60 – 69 | 13,875 | 29.7 | 1,310 | 41.3 | 695 | 40.2 | 5,880 | 29.3 | 352 | 39.9 | 5,798 | 31.9 | 273 | 37.2 |
|  | ≥ 70 | 15,484 | 33.2 | 1,241 | 39.2 | 728 | 42.1 | 8,053 | 40.2 | 411 | 46.6 | 6,961 | 38.3 | 349 | 47.6 |
| State of residence | |  |  |  |  |  |  |  |  |  |  |  |  |  |  |
|  | Iowa | 28,674 | 61.5 | 1,928 | 60.8 | 1,137 | 65.7 | 13,058 | 65.2 | 598 | 67.7 | 12,062 | 66.3 | 487 | 66.4 |
|  | North Carolina | 17,986 | 38.5 | 1,241 | 39.2 | 593 | 34.3 | 6,982 | 34.8 | 285 | 32.3 | 6,137 | 33.7 | 247 | 33.7 |
| Race | |  |  |  |  |  |  |  |  |  |  |  |  |  |  |
|  | White | 45,381 | 97.3 | 3,012 | 95.1 | 1,657 | 95.8 | 19,694 | 98.3 | 860 | 97.4 | 17,892 | 98.3 | 709 | 96.6 |
|  | Black, Other, Missing | 1,279 | 2.7 | 157 | 5.0 | 73 | 4.2 | 346 | 1.7 | 23 | 2.6 | 307 | 1.7 | 25 | 3.4 |
| First degree family history of PCa | |  |  |  |  |  |  |  |  |  |  |  |  |  |  |
|  | No | 38,494 | 82.5 | 2,348 | 74.1 | 1,312 | 75.8 | 16,685 | 83.3 | 688 | 77.9 | 15,262 | 83.9 | 566 | 77.1 |
|  | Yes | 3,529 | 7.6 | 455 | 14.4 | 239 | 13.8 | 1,675 | 8.4 | 125 | 14.2 | 1,525 | 8.4 | 106 | 14.4 |
|  | Missing | 4,637 | 9.9 | 366 | 11.6 | 179 | 10.4 | 1,680 | 8.4 | 70 | 7.9 | 1,412 | 7.8 | 62 | 8.5 |
| Smoking status | |  |  |  |  |  |  |  |  |  |  |  |  |  |  |
|  | Never | 23,974 | 51.4 | 1,552 | 49.0 | 861 | 49.8 | 10,466 | 52.2 | 446 | 50.5 | 9,893 | 54.4 | 387 | 52.7 |
|  | Former | 13,996 | 30.0 | 1,226 | 38.7 | 662 | 38.3 | 6,511 | 32.5 | 342 | 38.7 | 5,803 | 31.9 | 274 | 37.3 |
|  | Current | 7,551 | 16.2 | 323 | 10.2 | 169 | 9.8 | 2,669 | 13.3 | 78 | 8.8 | 2,421 | 13.3 | 68 | 9.3 |
|  | Missing | 1,139 | 2.4 | 68 | 2.2 | 38 | 2.2 | 394 | 2.0 | 17 | 1.9 | 82 | 0.5 | 5 | 0.7 |
| Age at diagnosis (years) | |  |  |  |  |  |  |  |  |  |  |  |  |  |  |
|  | < 60 | - | - | 618 | 19.5 | 307 | 17.8 | - | - | 120 | 13.6 | - | - | 112 | 15.3 |
|  | 60 – 64 | - | - | 561 | 17.7 | 303 | 17.5 | - | - | 146 | 16.5 | - | - | 106 | 14.4 |
|  | 65 – 69 | - | - | 749 | 23.6 | 392 | 22.7 | - | - | 206 | 23.3 | - | - | 167 | 22.8 |
|  | 70 – 74 | - | - | 623 | 19.7 | 341 | 19.7 | - | - | 189 | 21.4 | - | - | 154 | 21.0 |
|  | 75 – 79 | - | - | 392 | 12.4 | 226 | 13.1 | - | - | 127 | 14.4 | - | - | 122 | 16.6 |
|  | ≥ 80 | - | - | 226 | 7.1 | 161 | 9.3 | - | - | 95 | 10.8 | - | - | 73 | 10.0 |

^a^ Percentages may not add up, due to rounding

^b^ Excludes females, commercial applicators, spouses, those who moved out of state, and prevalent cancer cases

^c^ Excludes females, commercial applicators, spouses, those who moved out of state, prevalent cancer cases, and take-home questionnaire non-responders.

^d^ Excludes females, commercial applicators, spouses, those who moved out of state, prevalent cancer cases, and those that did not respond to both follow-up questionnaires.
