# Supplementary material for: Pesticide exposure and risk of aggressive prostate cancer among private pesticide applicators
Source: Environ Health. 2020 Mar 5;19:30. doi: 10.1186/s12940-020-00583-0 (PMC7059337; doi:10.1186/s12940-020-00583-0)
Supplement: Supplementary file 5 — Additional file 5: Table S5. Prostate specific antigen (PSA) frequencies by selected characteristicsa. [file 12940_2020_583_MOESM5_ESM.docx]

Supplemental Table 5. Prostate specific antigen (PSA) frequencies by selected characteristics^a^

| Characteristic | | Prostate specific antigen (PSA) checked  (n=28,880) | | | | |  |
| --- | --- | --- | --- | --- | --- | --- | --- |
|  |  | No  (n= 5464) | | Yes  (n=23416) | | OR (95% CI) |  |
|  |  | N | % | N | % |  |  |
| State | |  |  |  |  |  |  |
|  | Iowa | 3882 | 71.0 | 15372 | 65.6 | Ref |  |
|  | North Carolina | 1582 | 29.0 | 8044 | 34.4 | 1.23 (1.14, 1.34) |  |
| Family history of PCa | |  |  |  |  |  |  |
|  | No | 4804 | 87.9 | 19253 | 82.2 | Ref |  |
|  | Yes | 274 | 5.0 | 2333 | 10.0 | 1.72 (1.49, 1.98) |  |
| Attained age (years) | |  |  |  |  |  |  |
|  | < 40 | 497 | 9.1 | 58 | 0.2 | 0.04 (0.03, 0.05) |  |
|  | 40 – 49 | 1763 | 32.3 | 1141 | 4.9 | 0.21 (0.19, 0.23) |  |
|  | 50 – 59 | 1834 | 33.6 | 5567 | 23.8 | Ref |  |
|  | 60 – 69 | 727 | 13.3 | 7229 | 30.9 | 3.10 (2.82, 3.4) |  |
|  | ≥ 70 | 643 | 11.8 | 9421 | 40.2 | 4.92 (4.44, 5.46) |  |
| Marital Status | |  |  |  |  |  |  |
|  | Single | 490 | 9.0 | 946 | 4.0 | 0.59 (0.52, 0.66) |  |
|  | Married | 3699 | 67.7 | 16338 | 69.8 | Ref |  |
|  | Divorced | 201 | 3.7 | 519 | 2.2 | 0.87 (0.73, 1.04) |  |
|  | Widowed | 68 | 1.2 | 603 | 2.6 | 0.75 (0.58, 0.98) |  |
| Smoking status | |  |  |  |  |  |  |
|  | Never | 3482 | 63.7 | 12096 | 51.7 | Ref |  |
|  | Former | 1030 | 18.9 | 8267 | 35.3 | 1.19 (1.09, 1.30) |  |
|  | Current | 915 | 16.7 | 2881 | 12.3 | 0.75 (0.68, 0.83) |  |
| Still farming at P3 | |  |  |  |  |  |  |
|  | No | 397 | 7.3 | 2208 | 9.4 | Ref |  |
|  | Yes | 3505 | 64.1 | 13324 | 56.9 | 1.03 (0.91, 1.18) |  |
| Education | |  |  |  |  |  |  |
|  | Less than high school | 2933 | 53.7 | 12268 | 52.4 | Ref |  |
|  | More than high school | 2347 | 43.0 | 10306 | 44.0 | 1.48 (1.37, 1.59) |  |
| Race | |  |  |  |  |  |  |
|  | White | 5385 | 98.6 | 22960 | 98.1 | Ref |  |
|  | Black, Other, Missing | 79 | 1.4 | 456 | 1.9 | 1.11 (0.84, 1.47) |  |
| Body Mass Index (BMI) | |  |  |  |  |  |  |
|  | Underweight | 15 | 0.3 | 27 | 0.1 | 0.81 (0.36, 1.83) |  |
|  | Normal | 1599 | 29.3 | 5092 | 21.7 | Ref |  |
|  | Overweight | 2371 | 43.4 | 11393 | 48.7 | 1.24 (1.14, 1.35) |  |
|  | Obese | 1019 | 18.6 | 5223 | 22.3 | 1.32 (1.19, 1.46) |  |
| Dimethoate | |  |  |  |  |  |  |
|  | Never | 2590 | 47.4 | 12683 | 54.2 | Ref |  |
|  | Ever | 85 | 1.56 | 579 | 2.5 | 1.10 (0.84, 1.42) |  |
| Triclopyr | |  |  |  |  |  |  |
|  | Never | 3467 | 63.5 | 14594 | 62.3 | Ref |  |
|  | Ever | 303 | 5.5 | 1175 | 5.0 | 1.15 (0.99, 1.35) |  |

^a^ Numbers may not sum to 1, due to missing; dimethoate and triclopyr frequencies may not add to total due to missing
